# Squeezing dynamics of a nanowire system with spin-orbit interaction

**DOI:** 10.1038/s41598-018-28607-3

**Published:** 2018-07-11

**Authors:** R. I. Mohamed, Ahmed Farouk, A. H. Homid, O. H. El-Kalaawy, Abdel-Haleem Abdel-Aty, M. Abdel-Aty, S. Ghose

**Affiliations:** 1 Department of Mathematics and Computer Science, Faculty of Science, Beni-Suef University, Beni-Suef, 62511 Egypt; 2 Department of Physics and Computer Science, Wilfrid Laurier University, Waterloo, Canada; 3 University of Science and Technology, Zewail City for Science and Technology, Giza, Egypt; 4 Faculty of science, Al-Azhar University, Assiut, 71524 Egypt; 5 Department of Mathematics, Faculty of Science, Sohag University, Sohag, Egypt; 6 Deanship of Scientific Research and Graduate Studies, Applied Science University, Sitra, Bahrain

## Abstract

We analyze the dynamics of squeezing in a ballistic quantum wire with Rashba spin-orbit interaction in the presence of both strong and weak magnetic fields and for different initial states of the system. Compared to the more standard measure of squeezing based on variances, we show that entropy squeezing is a more sensitive measure. Our results show that there is a strong relationship between the spin-orbit interaction and the strength of entropy squeezing. Furthermore, there is a relationship between the initial state and the number of squeezed components. This allows new knobs to control the strength and the component of entropy squeezing in a nanowire system.

## Introduction

Many recent studies have focused on the physical properties of semiconductor quantum wires because of their potential technological applications and for building quantum computing devices^1–3^. Progress towards the use of one dimensional nanostructures in an extensive range of prospective nanoscale devices applications and complex functional architectures has been reviewed in^4^. Various metals/alloys, semiconductors have been explored for nanodevices fabrication using doped semiconducting nanowires^5^. The electronic transport properties of semiconductor nano-objects and their applications for quantum information processing have been explored^6^. Furthermore, the optical properties of quantum dots and wires have been discussed^7^. Past work^8^ has also explored the effect of a magnetic field on the spectral and spin properties of a ballistic quasi-one-dimensional electron system with Rashba effect^9^. These studies have shown the potential of nanowire systems for quantum computing applications. In this paper, we therefore study a nanowire system from a quantum information theoretic perspective. Our objective is to explore the behaviour of squeezing in a new system consisting of a nanowire with Rashba spin-orbit interaction in the presence of strong and weak magnetic fields for potential applications in quantum information processing.

Previous studies have explored the effect of magnetic fields on nanowires properties, but have not studied their effect on squeezing of the quantum states. The spectral, transport and conductance properties of ballistic quasi-one-dimensional systems in the presence of spin-orbit coupling and in-plane magnetic fields has been previously analyzed^10,11^. Calculations of the effective *g* factor of conduction electrons in nanowires subjected to in-plane magnetic fields in the presence of Rashba and Dresselhaus spin-orbit interactions was discussed in^12^. The electronic structure, spin and transport properties of double quantum wires subjected to an in-plane magnetic field by taking into account Rashba and Dresselhaus spin-orbit couplings has also been investigated^13^. Furthermore, it was shown that magnetic field effects on spin texturing in a quantum wire with Rashba spin-orbit interaction introduce additional complex features in spin texturing^14^. In this paper we add to the growing body of literature on nanowires interacting with magnetic fields by examining the dynamics of squeezing in the presence of magnetic fields.

Recent work has focused on atomic squeezing for its potential applications in quantum information theory. For instance, some applications of atom squeezing are in quantum teleportation, cryptography, and dense coding^15–18^. Atomic squeezing is based on the Heisenberg uncertainty relation (HUR)^19^, which provides a lower bound on quantum fluctuations. The HUR is formulated in terms of the variance (standard deviations) for the system and is not the optimal measure of information squeezing in some circumstances. An alternative approach to quantifying information squeezing is via the entropic uncertainty relation (EUR)^20–24^. The EUR has been applied to study entropy squeezing in various systems including the Jaynes-Cummings model and its generalizations^25–28^. Here we compare entropy squeezing and variance squeezing in a nanowire with Rashba spin-orbit interaction interacting with strong or weak magnetic fields. We calculate the evolutions of the entropy and variance squeezing for different initial states of the system and for different strengths of magnetic field. Our results show that entropy squeezing is more sensitive to changes in the initial state and magnetic field compared to spin squeezing. The spin-orbit interaction strongly effects the strength and oscillation frequency of the dynamics of entropy squeezing in both strong and weak magnetic fields. The initial state effects which component (quadrature) is squeezed. Our results thus identify ways to control both the strength and the quadrature of squeezing in the system. The article is organized as follows; in section 2, the Hamiltonian model and the derivation of the time evolution of the density operator of the system are described. Expressions for the evolution of variance and entropy squeezing for the proposed model are calculated in section 3. The squeezing dynamics of the system for different system properties, and initial conditions are discussed in section 4. Finally, section 5 includes a summary and conclusion.

## The Hamiltonian Model

In our proposed model, we consider a ballistic quantum wire with Rashba spin-orbit interaction due to the structural inversion asymmetry in a two-dimensional electron gas such as InAs presented in^8^. In the presence of a perpendicular magnetic field, we assume that the wire axis is taken along the y-direction with a parabolic lateral confining potential in the x-direction. Moreover, the external electric field $\overrightarrow{F}$ is applied along the direction of quantum confinement to generate a Stark shift in the electron spectra. Furthermore, top gates are utilized for controlling the strength of the Rashba interaction^29^.

We first briefly review the Hamiltonian for perpendicular magnetic fields^30,31^. The Hamiltonian can be written as

1 $$H=\frac{{(\overrightarrow{P}+e\overrightarrow{A})}^{2}}{2m}+\frac{1}{2}m{\omega }_{o}^{2}{x}^{2}+eFx+\frac{1}{2}g{\mu }_{B}\overrightarrow{\sigma }\mathrm{.}\overrightarrow{B}+\frac{{\alpha }_{r}}{\hslash }{[\overrightarrow{\sigma }\times (\overrightarrow{P}+e\overrightarrow{A})]}_{z},$$

where *m* and *ω*_*o*_ are the effective mass and characteristic frequency of the parabolic confinement, $\overrightarrow{P}=({p}_{x},\,{p}_{y})$ is the linear momentum, $\overrightarrow{\sigma }=({\sigma }_{x},\,{\sigma }_{y},\,{\sigma }_{z})$ is the Pauli vector operator, *g* is Lande’s *g*-factor, and *μ*_*B*_ is the Bohr magneton. The perpendicular magnetic field $\overrightarrow{B}=\mathrm{(0,}\,\mathrm{0,}\,B)$ corresponds to the vector potential $\overrightarrow{A}=xB{\hat{e}}_{y}$ in the Landau gauge. The last term in Eq. (1) represents the Rashba spin-orbit interaction (RSOI), where *α*_*r*_ is the Rashba spin-orbit interaction parameter.

To simplify the Hamiltonian model, three length scales to characterize the strength of the lateral confining potential, magnetic field *B* and SOI are introduced,

2 $${l}_{o}=\sqrt{\frac{\hslash }{m{\omega }_{o}}}\,\,{l}_{B}=\sqrt{\frac{\hslash }{m{\omega }_{c}}}\,\,{l}_{so}=\frac{{\hslash }^{2}}{2m{\alpha }_{r}},$$

where, the length scale *l*_*o*_ corresponds to the confinement potential, *l*_*B*_ is the magnetic length with *ω*_*c*_ = *eB*/*m* the cyclotron frequency, and *l*_*so*_ is the length scale associated with the SOI. Furthermore, ${K}_{F}=\frac{eF}{\hslash {\omega }_{o}}$ can be used to characterize the action of the external electric field on electrons.

Since the Hamiltonian is translationally invariant along the *y*-direction, the momentum component *p*_*y*_ can be replaced by $\hslash k$. Then, the Hamiltonian in units of $\hslash {\omega }_{o}$ for a given *k* can be expressed, by using creation and annihilation operators of a shifted harmonic oscillator, ${a}^{\dagger }$ and *a*, in the following dimensionless form:

3 $$H={H}_{o}+H^{\prime} ,$$

where

4 $${H}_{o}={\rm{\Omega }}({a}^{\dagger }a+\frac{1}{2})+\frac{1}{2}{({l}_{o}k)}^{2}-\frac{1}{2}{(\frac{{\rm{\Omega }}{\chi }_{c}}{{l}_{o}})}^{2}+\frac{1}{2}{\xi }_{1}{\sigma }_{x}+\frac{1}{2}\delta {\sigma }_{z},$$

and

5 $$H^{\prime} =\frac{1}{2}{\xi }_{2}({a}^{\dagger }+a){\sigma }_{x}+\frac{i}{2}{\xi }_{3}(a-{a}^{\dagger }){\sigma }_{y}\mathrm{.}$$

The parameters in the above equations are defined as

6 $$\begin{array}{c}{\rm{\Omega }}=\sqrt{1+{(\frac{{l}_{o}}{{l}_{B}})}^{4}}=\frac{\sqrt{{\omega }_{o}^{2}+{\omega }_{c}^{2}}}{{\omega }_{o}},\,\,{\chi }_{c}=\frac{{l}_{o}}{{{\rm{\Omega }}}^{2}}[{l}_{o}{K}_{F}+{l}_{o}k{(\frac{{l}_{o}}{{l}_{B}})}^{2}],\\ {\xi }_{1}=\frac{{l}_{o}}{{l}_{so}}({l}_{o}k-\frac{{l}_{o}{\chi }_{c}}{{l}_{B}^{2}}),\,\,{\xi }_{2}=\frac{1}{\sqrt{2}}\frac{{l}_{o}}{{l}_{so}}{(\frac{{l}_{o}}{{l}_{B}})}^{2}\frac{1}{\sqrt{{\rm{\Omega }}}},\,\,{\xi }_{3}=\sqrt{\frac{{\rm{\Omega }}}{2}}\frac{{l}_{o}}{{l}_{so}},\end{array}$$

and the dimensionless Zeeman splitting $\delta =\frac{g}{2}{(\frac{{l}_{o}}{{l}_{B}})}^{2}\frac{m}{{m}_{o}}$ is given in terms of the free electron mass *m*_*o*_. The spin-orbit interaction in *H*′ leads to coupling of neighboring energy subbands. Also, the presence of the magnetic field produces a lateral shift in the wave function and the renormalization of the oscillator frequency Ω.

In the absence of an external electric field and *kl*_*o*_ ≪ 1, we have *ξ*_1_ = 0, and in the case of a strong magnetic field (*l*_*B*_ ≪ *l*_*o*_), Eq. (3) becomes the exactly integrable Jaynes-Cummings model^16^ in the rotating-wave approximation^8^,

7 $$\frac{H}{\hslash {\omega }_{c}}=({a}^{\dagger }a+\frac{1}{2})+\frac{1}{4}\frac{m}{{m}_{o}}g{\sigma }_{z}+\frac{1}{\sqrt{2}}\frac{{l}_{B}}{{l}_{so}}({a}^{\dagger }{\sigma }_{-}+a{\sigma }_{+}\mathrm{).}$$

In our work, a nanowire is equivalent to a two-qubit system. So, we can assume that the electrons occupy the lowest energy state {|0, ↑〉,|1, ↓〉} which consists of the spin degenerate ground and first excited eigenstates related to the confinement in the *x* direction.

We consider that the energy state of spin is initially in a superposition state

8 $$|{\rm{\Psi }}\mathrm{(0)}\rangle =\,\cos (\frac{\theta }{2})|\mathrm{0,}\uparrow \rangle +\,\sin (\frac{\theta }{2}){e}^{-i\varphi }|\mathrm{1,}\downarrow \rangle \mathrm{.}$$

The corresponding initial density operator at *t* = 0 is

9 $$\hat{\rho }\mathrm{(0)}=({\cos }^{2}(\frac{\theta }{2})|0,\,\uparrow \rangle \langle \uparrow ,\,0|+{\sin }^{2}(\frac{\theta }{2})|\mathrm{1,}\,\downarrow \rangle \langle \downarrow ,\,1|+\frac{1}{2}\,\sin (\theta )({e}^{i\varphi }|\mathrm{0,}\,\uparrow \rangle \langle \downarrow ,\,1|+{e}^{-i\varphi }|\mathrm{1,}\,\downarrow \rangle \langle \uparrow ,\,0|))\mathrm{.}$$

The density operator of the proposed model for *t* > 0 is then

10 $$\hat{\rho }(t)={C}_{o}{C}_{o}^{\ast }|\mathrm{0,}\,\uparrow \rangle \langle \uparrow ,\,0|+{C}_{1}{C}_{1}^{\ast }|\mathrm{1,}\,\downarrow \rangle \langle \downarrow ,\,1|+{C}_{o}{C}_{1}^{\ast }|\mathrm{0,}\,\uparrow \rangle \langle \downarrow ,\,1|+{C}_{1}{C}_{o}^{\ast }|\mathrm{1,}\,\downarrow \rangle \langle \uparrow ,\,0|,$$

where *C*_*o*_(*t*) and *C*_1_(*t*) can be written as

11 $${C}_{0}(t)={e}^{-it}[\cos (\theta /\mathrm{2)}(\cos \,ut-i\eta \frac{sin\,ut}{u})-i\lambda \,\sin (\theta \mathrm{/2}){e}^{-i\varphi }\frac{\sin \,ut}{u}]$$

12 $${C}_{1}(t)={e}^{-it}[\sin (\theta \mathrm{/2)}{e}^{-i\varphi }(\cos \,ut+i\eta \frac{\sin \,ut}{u})-i\lambda \,\cos (\theta \mathrm{/2)}\frac{\sin \,ut}{u}]\mathrm{.}$$

The quantities *u*, *λ* and *η* in the above equations are

13 $$u=\sqrt{{\eta }^{2}+{\lambda }^{2}}\,\,\lambda =\frac{1}{\sqrt{2}}\frac{{l}_{B}}{{l}_{so}}\,\,\eta =\frac{1}{2}(\nu -\mathrm{1)}\,\,\nu =\frac{1}{2}\frac{m}{{m}_{o}}g\mathrm{.}$$

In the following section we use the above equations for the evolution of the density operator to study entropy squeezing.

## Variance and Entropy Squeezing

The study of information squeezing depends on the Heisenberg uncertainty relation^20^. For any two hermitian operators $\hat{N}$ and $\hat{M}$ complying with the commutation correlation $[\hat{N},\,\hat{M}]=i\hat{T}$, the Heisenberg uncertainty relation states that ${\rm{\Delta }}\hat{N}{\rm{\Delta }}\hat{M}\ge \frac{1}{2}|\langle \hat{T}\rangle |$ where the variance ${\rm{\Delta }}\hat{N}=\sqrt{\langle {\hat{N}}^{2}\rangle -{\langle \hat{N}\rangle }^{2}}$. For a two-level system described by the Pauli operators ${\hat{S}}_{x}$, ${\hat{S}}_{y}$, and ${\hat{S}}_{z}$ satisfying $[{\hat{S}}_{x},\,{\hat{S}}_{y}]=i{\hat{S}}_{z}$, the Heisenberg uncertainty principle can be written as ${\rm{\Delta }}{\hat{S}}_{x}{\rm{\Delta }}{\hat{S}}_{y}\ge \frac{1}{2}|\langle {\hat{S}}_{z}\rangle |$^32^. The variance in the component ${\hat{S}}_{\alpha }$ of the dipole of a two-level state is squeezed if ${\hat{S}}_{\alpha }$ fulfills the following prerequisite:

14 $$V({\hat{S}}_{\alpha })=({\rm{\Delta }}{\hat{S}}_{\alpha }-\sqrt{|\langle {\hat{S}}_{z}\rangle \mathrm{/2|}}) < \mathrm{0,}\,\,\alpha =x,\,y\mathrm{.}$$

We can define the entropy squeezing for a two-level energy state by using the quantum information theoretic measure of entropy^25–27^. The information entropy is defined as

15 $$H({S}_{\alpha })=-\,\sum _{i=1}^{2}{P}_{i}({S}_{\alpha })\,\mathrm{ln}\,{P}_{i}({S}_{\alpha }),\,\,\,\,\alpha =(x,\,y,\,z),$$

where *P*_*i*_(*S*_*α*_) = 〈*ψ*_*αi*_|*ρ*|*ψ*_*αi*_〉,(*i* = 1, 2), is the probability distribution of operator ${\hat{S}}_{\alpha }$ and |*ψ*_*αi*_〉 is the eigenstate of the operator ${\hat{S}}_{\alpha }$, where ${\hat{S}}_{\alpha }|{\psi }_{\alpha i}\rangle ={\lambda }_{\alpha i}|{\psi }_{\alpha i}\rangle$. For a two-level state the entropic uncertainty relation is derived as

16 $$H({S}_{x})+H({S}_{y})+H({S}_{z})\ge 2\,\mathrm{ln}\,2.$$

Eq. (16) can be used to obtain the relation

17 $$\delta H({S}_{x})\delta H({S}_{y})\ge \frac{4}{\delta H({S}_{z})},$$

where *δH*(*S*_*α*_) = exp[*H*(*S*_*α*_]. The fluctuations in component *S*_*α*_(*α* = *x*, *y*) can be “squeezed in entropy” if the information entropy *H*(*S*_*α*_) of *S*_*α*_ fulfills the following prerequisite,

18 $$E({S}_{\alpha })=(\delta H({S}_{\alpha })-\frac{2}{\sqrt{|\delta H({S}_{z})|}}) < 0.$$

By using the density operator *ρ*(*t*), we can write the information entropies of the component operators *S*_*x*_, *S*_*y*_ and *S*_*z*_ in the form

19 $$H({S}_{x})=-[\frac{1}{2}+Re\{{\rho }_{12}(t)\}]\,\mathrm{ln}\,[\frac{1}{2}+Re\{{\rho }_{12}(t)\}]-[\frac{1}{2}-Re\{{\rho }_{12}(t)\}]\,\mathrm{ln}\,[\frac{1}{2}-Re\{{\rho }_{12}(t)\}],$$

20 $$H({S}_{y})=-[\frac{1}{2}+Im\{{\rho }_{12}(t)\}]\,\mathrm{ln}\,[\frac{1}{2}+Im\{{\rho }_{12}(t)\}]-[\frac{1}{2}-Im\{{\rho }_{12}(t)\}]\,\mathrm{ln}\,[\frac{1}{2}-Im\{{\rho }_{12}(t)\}],$$

21 $$H({S}_{z})=-\,{\rho }_{11}(t)\,\mathrm{ln}\,{\rho }_{11}(t)-{\rho }_{22}(t)\,\mathrm{ln}\,{\rho }_{22}(t),$$

where *ρ*_12_(*t*), *ρ*_11_(*t*), *ρ*_22_(*t*) and ${\rho }_{21}(t)={({\rho }_{12}(t))}^{\dagger }$ are calculated from the following equations,

22 $$\begin{array}{rcl}{\rho }_{11}(t) & = & {\cos }^{{\rm{2}}}(\theta /\mathrm{2)}[{\cos }^{2}ut+{(\frac{\eta \sin ut}{u})}^{2}]+{\lambda }^{2}{\sin }^{{\rm{2}}}(\theta \mathrm{/2)}{(\frac{\sin ut}{u})}^{2}\\  &  & -\,\lambda \,\sin \,\theta \frac{\sin \,ut}{u}(\cos \,ut\,\sin \,\phi -\eta \,\cos \,\phi \frac{\sin \,ut}{u}),\end{array}$$

23 $$\begin{array}{rcl}{\rho }_{22}(t) & = & si{n}^{2}(\theta \mathrm{/2)}[{\cos }^{{\rm{2}}}ut+{(\frac{\eta \sin ut}{u})}^{2}]+{\lambda }^{2}{\cos }^{{\rm{2}}}(\theta \mathrm{/2)}{(\frac{\sin ut}{u})}^{2}\\  &  & +\,\lambda \,\sin \,\theta \frac{\sin \,ut}{u}(\cos \,ut\,\sin \,\phi -\eta \,\cos \,\phi \frac{\sin \,ut}{u}),\end{array}$$

and

24 $${\rho }_{12}(t)=R(t)+iV(t\mathrm{).}$$

Here *R*(*t*) and *V*(*t*) are given by

25 $$\begin{array}{rcl}R(t) & = & \frac{1}{2}\,\sin \,\theta \,\cos \,\phi [{\cos }^{2}ut-{(\frac{\eta \sin ut}{u})}^{2}]+\frac{1}{2}{\lambda }^{2}\,\sin \,\theta \,\cos \,\phi {(\frac{\sin ut}{u})}^{2}\\  &  & +\,\lambda \eta \,\cos \,\theta {(\frac{\sin ut}{u})}^{2}+\eta \,\sin \,\theta \,\sin \,\phi \,\cos \,ut(\frac{\sin \,ut}{u})\end{array}$$

26 $$\begin{array}{rcl}V(t) & = & \frac{1}{2}\,\sin \,\theta \,\sin \,\phi [{\cos }^{2}ut-{(\frac{\eta \sin ut}{u})}^{2}]-\frac{1}{2}{\lambda }^{2}\,\sin \,\theta \,\sin \,\phi {(\frac{\sin ut}{u})}^{2}\\  &  & +\,\lambda \,\cos \,\theta \,\cos \,ut(\frac{\sin \,ut}{u})-\eta \,\sin \,\theta \,\cos \,\phi \,\cos \,ut(\frac{\sin \,ut}{u})\end{array}$$

## Results and Discussion

We now discuss the effects of the initial state and spin-orbit interaction strength on entropy squeezing versus variance squeezing for a quantum wire system with different strengths of the magnetic field.

### Effect of the Initial State on Squeezing in a Strong Magnetic Field (*l*_*B*_ ≪ *l*_*o*_)

**Figure 1:**
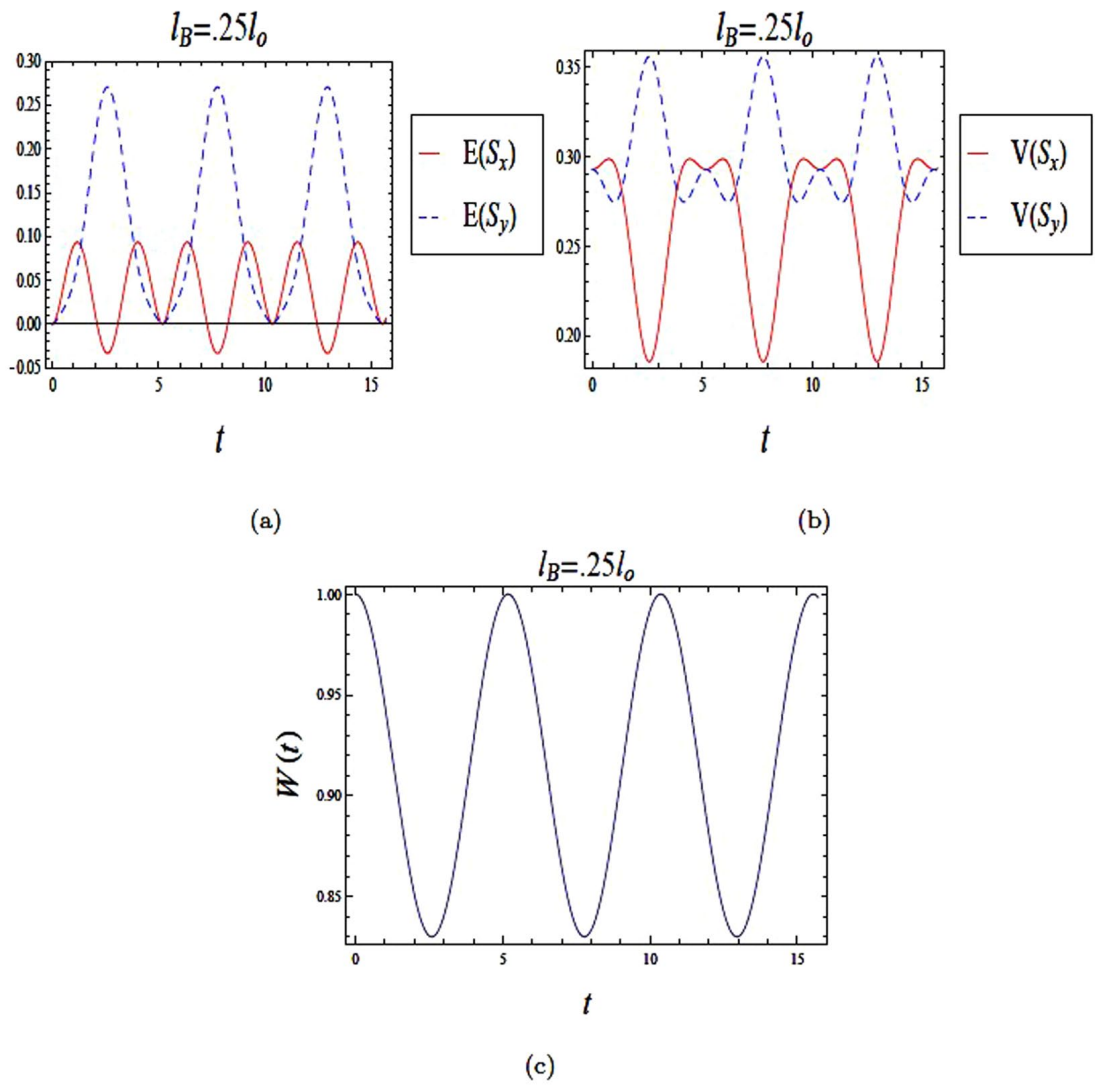
The time evolution of entropy and variance squeezing for a quantum wire with SOI in a strong magnetic field with *l*_*B*_ = 0.25*l*_*o*_ and *l*_*so*_ = *l*_*o*_. The initial state is an excited state, such that *θ* = 0 and the relative phase *ϕ* = 0. (**a**) Entropy squeezing factors *E*(*S*_*x*_) and *E*(*S*_*y*_); (**b**)Variance squeezing factors *V*(*S*_*x*_) and *V*(*S*_*y*_); (**c**) Qubit inversion.

In our computation, we consider InAs with *α*_*r*_ = 1.0 × 10^−11^ *eVm*, *g* = −8, *m* = 0.04*m*_*o*_ and *l*_*so*_ = *l*_*o*_. In Fig. (1), we plot the time evolution of entropy squeezing and variance squeezing for a quantum wire with SOI in a strong magnetic field *l*_*B*_ = 0.25*l*_*o*_ and *l*_*so*_ = *l*_*o*_. The initial state corresponds to an excited state with *θ* = 0 and the relative phase *ϕ* = 0. In Fig. 1(a), we observe that there is squeezing in *E*(*S*_*x*_), while no squeezing occurs in the other quadrature *E*(*S*_*y*_). In contrast, it is clear from Fig. 1(b) that no squeezing occurs in either variance *V*(*S*_*x*_) or *V*(*S*_*y*_). Furthermore, the qubit inversion *W*(*t*)^33^, which is defined as the difference between the final state (|*C*_1_(*t*)|^2)^and the initial state (|*C*_0_(*t*)|^2^) is plotted in Fig. 1(c). From Fig. (1), we can conclude that the information entropies have more information than the variances of the two-level energy state. Moreover, it shows that entropy squeezing factors is a best measure of information squeezing state than variance factors.

**Figure 2:**
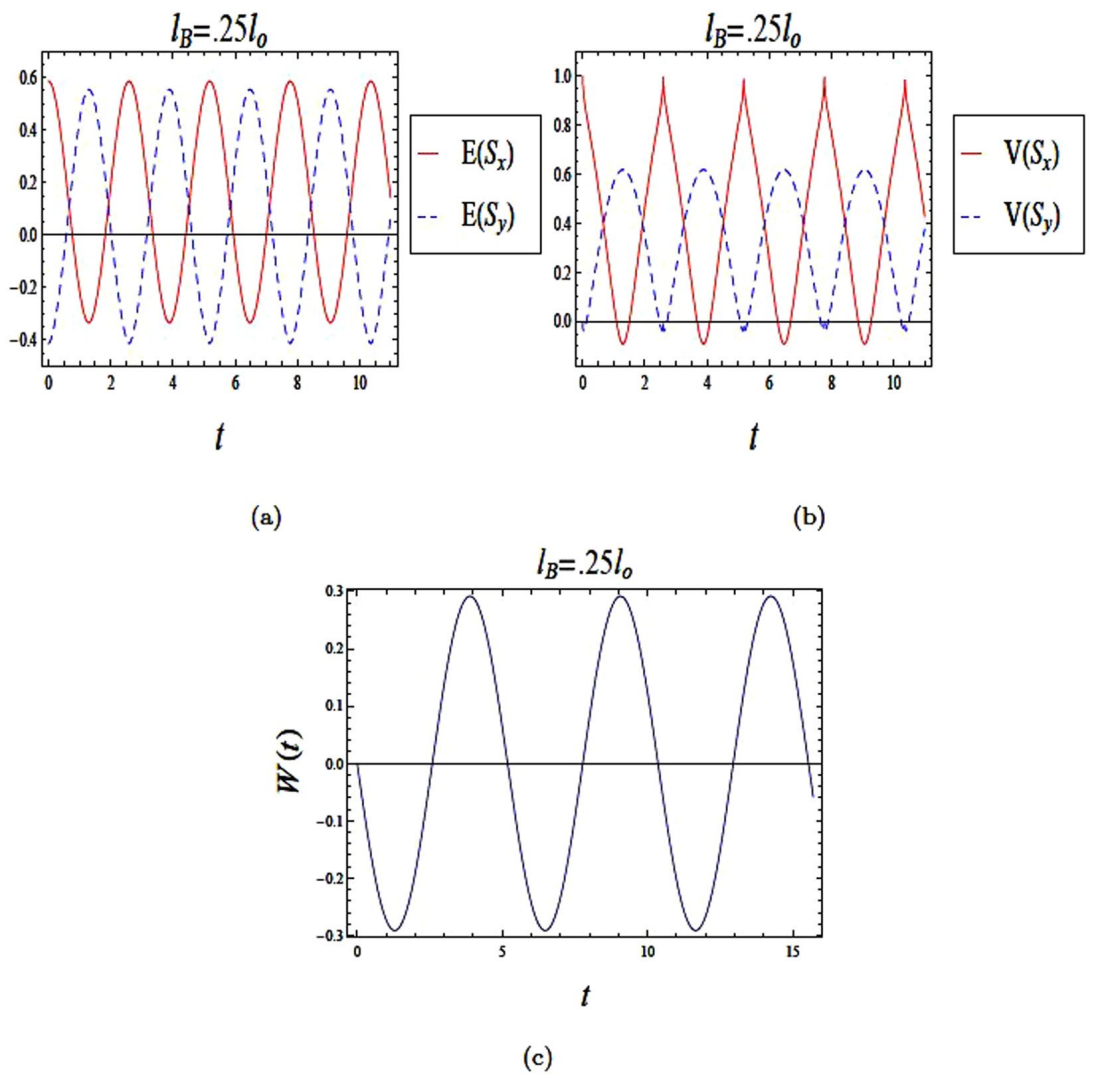
The time evolution of entropy and variance squeezing for a quantum wire with SOI in a strong magnetic field with *l*_*B*_ = 0.25*l*_*o*_ and *l*_*so*_ = *l*_*o*_. The initial state is a superposition state, such that *θ* = *π*/2 and the relative phase *ϕ* = *π*/2. (**a**) Entropy squeezing factors *E*(*S*_*x*_) and *E*(*S*_*y*_); (**b**) Variance squeezing factors *V*(*S*_*x*_) and *V*(*S*_*y*_); (**c**) Qubit inversion.

We study the effect of an initial superposition state on the entropy squeezing and variance squeezing in a strong magnetic field in Fig. 2. We start with an initial superposition such that *θ* = *π*/2 and the relative phase *ϕ* = *π*/2. In Fig. 2, squeezing of both entropy and variance are clearly observed. However, there are differences between entropy squeezing *E*(*S*_*x*,*y*_) and variance squeezing *V*(*S*_*x*,*y*_). From Fig. (2), the amount of squeezing *E*(*S*_*x*,*y*_) is greater than *V*(*S*_*x*,*y*_). Therefore, the information entropy appears to be a more sensitive measure of squeezing. Comparing Figs (1) and (2), we also note that if the evolution starts with an excited state, the squeezing occurs only within one component. On the other hand, when the initial state is a superposition, squeezing occurs in both components.

### Effect of the Initial State on Squeezing in a Weak Magnetic Field (*l*_*B*_ ≫ *l*_*o*_)

Our computations for a weak magnetic field are similar to those for the strong magnetic field with slight differences achieved by using the following numerical parameters,

$$u=\sqrt{{\eta }^{2}+{\lambda }^{2}}\,\,\lambda =\frac{1}{2}({\xi }_{2}+{\xi }_{3})\,\,\eta =\frac{1}{2}(\delta -{\rm{\Omega }}\mathrm{).}$$

**Figure 3:**
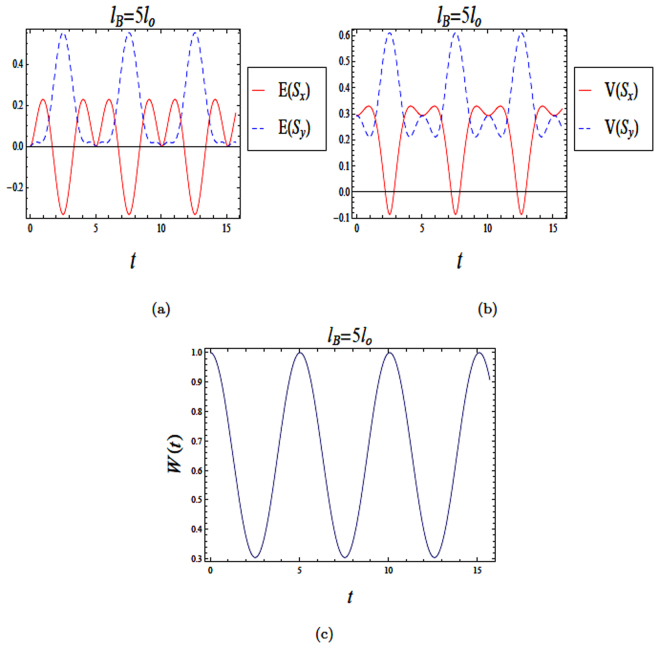
The time evolution of entropy and variance squeezing for a quantum wire with SOI in a weak magnetic field with *l*_*B*_ = 5*l*_*o*_ and *l*_*so*_ = *l*_*o*_. The initial state is an excited state, such that *θ* = 0 and the relative phase *ϕ* = 0. (**a**) Entropy squeezing factors *E*(*S*_*x*_) and *E*(*S*_*y*_); (**b**) Variance squeezing factors *V*(*S*_*x*_) and *V*(*S*_*y*_); (**c**) Qubit inversion.

**Figure 4:**
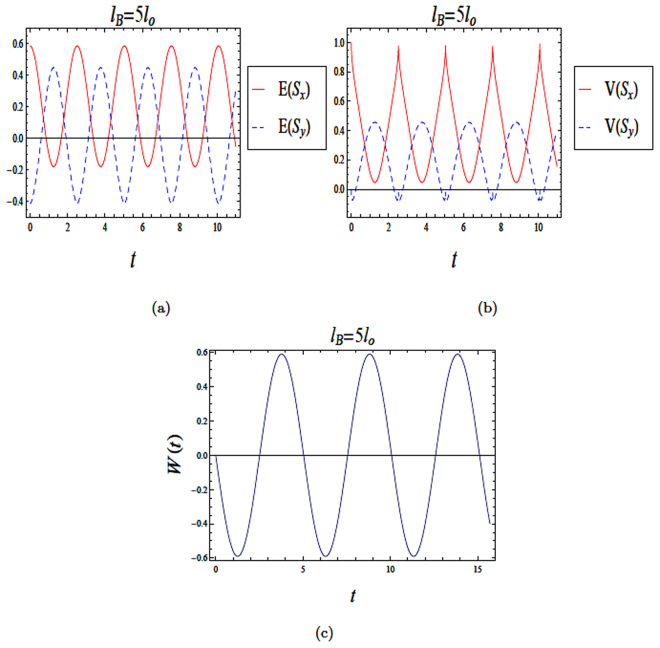
The time evolution of entropy and variance squeezing for a quantum wire with SOI in a weak magnetic field with *l*_*B*_ = 5*l*_*o*_ and *l*_*so*_ = *l*_*o*_. The initial state is a superposition state, such that *θ* = *π*/2 and the relative phase *ϕ* = *π*/2. (**a**) Entropy squeezing factors *E*(*S*_*x*_) and *E*(*S*_*y*_); (**b**) Variance squeezing factors *V*(*S*_*x*_) and *V*(*S*_*y*_); (**c**) Qubit inversion.

Figure 3 shows the time evolutions of both entropy squeezing and variance squeezing for a quantum wire with SOI in a weak magnetic field with *l*_*B*_ = 5*l*_*o*_ and *l*_*so*_ = *l*_*o*_. The initial states in the excited state. From Fig. 3(a), squeezing occurs in *E*(*S*_*x*_) but not in *E*(*S*_*y*_). Similarly, squeezing in *V*(*S*_*x*_) is observed, while no squeezing occurs in *V*(*S*_*y*_) as shown in Fig. 3(b). From Fig. 3, we can conclude that the amount of squeezing in *E*(*S*_*x*_) is greater than in the variance *V*(*S*_*x*_). For a weak magnetic field *l*_*B*_ = 5*l*_*o*_, if the system starts in a superposition state *θ* = *π*/2 and *ϕ* = *π*/2, then squeezing occurs in both *E*(*S*_*x*_) and *E*(*S*_*y*_)as shown in Fig. 4(a). In contrast, Fig. 4(b) shows that squeezing occurs only in the variance *V*(*S*_*x*_). To sum up, the entropy squeezing is a more sensitive measure of squeezing than variance

### Effect of Spin-orbit Interaction Strength (*l*_*so*_) on Squeezing in both Strong and Weak Magnetic Fields

**Figure 5:**
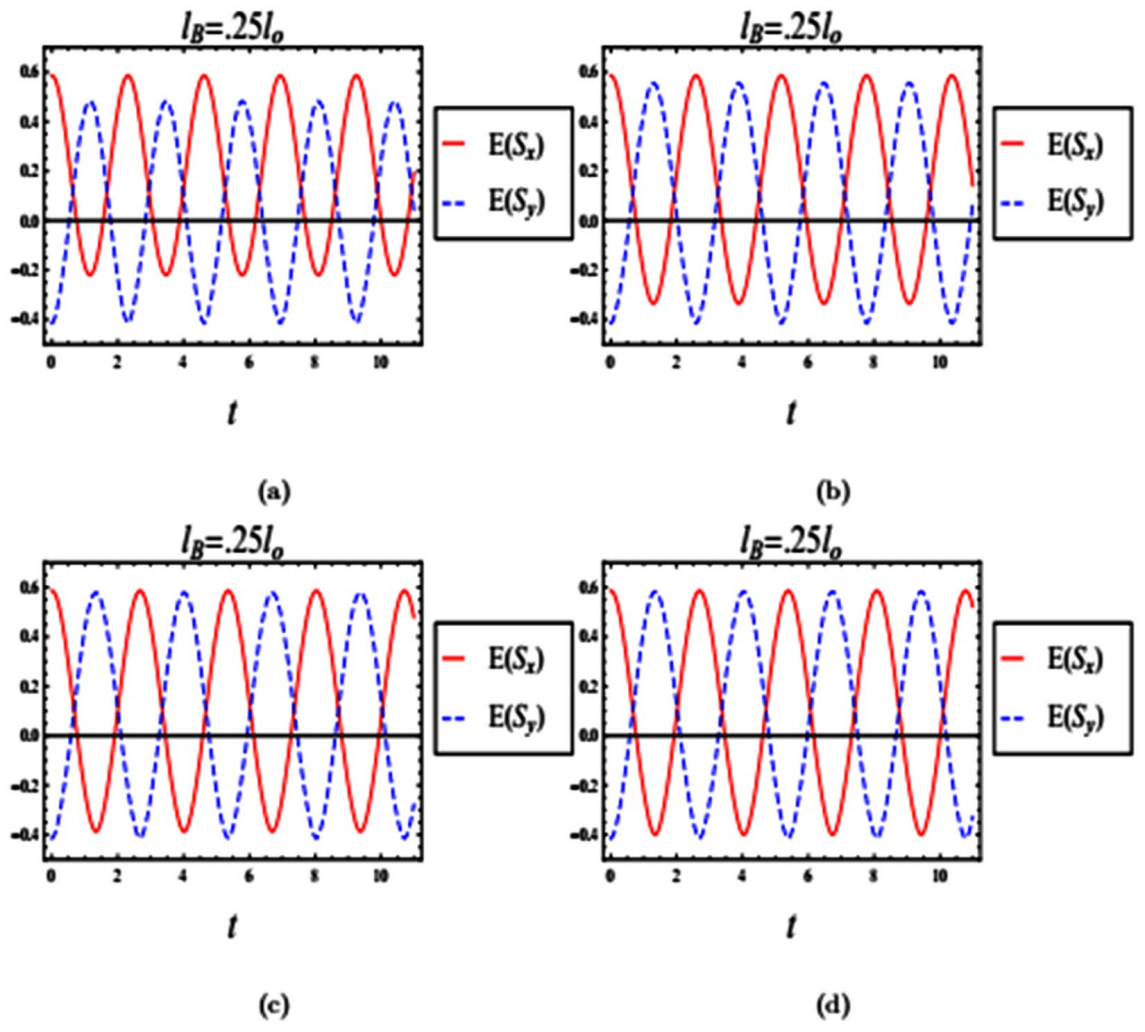
The time evolution of entropy squeezing *E*(*S*_*x*_) and *E*(*S*_*y*_) for a quantum wire with SOI in a strong magnetic field with *l*_*B*_ = 0.25*l*_*o*_. The initial states is a superposition state with *θ* = *π*/2 and the relative phase *ϕ* = *π*/2. (**a**) *l*_*so*_ = 0.5*l*_*o*_; (**b**) *l*_*so*_ = *l*_*o*_; (**c**) *l*_*so*_ = 2*l*_*o*_; (**d**) *l*_*so*_ = 3*l*_*o*_.

**Figure 6:**
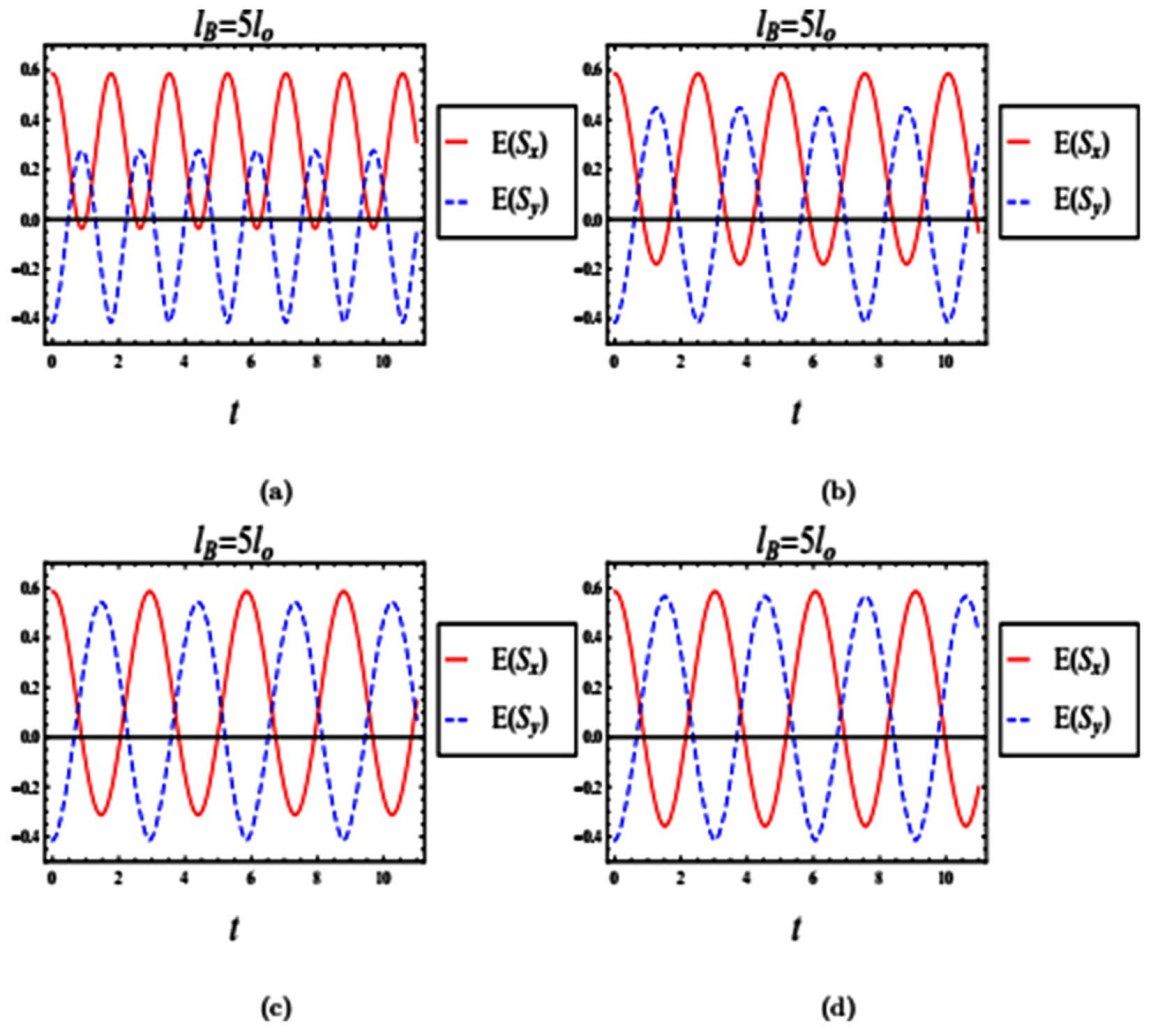
The time evolution of entropy squeezing *E*(*S*_*x*_) and *E*(*S*_*y*_) for a quantum wire with SOI in a weak magnetic field with *l*_*B*_ = 5*l*_*o*_. The initial states is a superposition state with *θ* = *π*/2 and relative phase *ϕ* = *π*/2. (**a**) *l*_*so*_ = 0.5*l*_*o*_; (**b**) *l*_*so*_ = *l*_*o*_; (**c**) *l*_*so*_ = 2*l*_*o*_; (**d**) *l*_*so*_ = 3*l*_*o*_.

The Spin-orbit Interaction strength (*l*_*so*_) influences the oscillation frequencies and the strength of entropy squeezing in both strong and weak magnetic fields. Figures (5) and (6) show the time evolutions of entropy squeezing factors *E*(*S*_*x*_) and *E*(*S*_*y*_) for our quantum wire system in a strong magnetic field (*l*_*B*_ = 0.25*l*_*o*_) and weak magnetic field (*l*_*B*_ = 5*l*_*o*_) respectively, as the spin-orbit interaction strength is varied. The initial state is a superposition state with *θ* = *π*/2 and *ϕ* = *π*/2. We can see that the entropy squeezing factors *E*(*S*_*x*_) and *E*(*S*_*y*_) periodically oscillate with a phase difference of *π*/2 and their amplitudes change as the spin-orbit interaction strength varied. When the spin-orbit interaction strength (*l*_*so*_) increased, the amount of entropy squeezing is increased and the number of periodic oscillations disappeared gradually.

### Time evolution of Squeezing in the Absence of a Magnetic Field (*B* = 0)

We now discuss the influence of spin-orbit interaction strength and the initial state of the system on the entropy squeezing in a quantum wire when the magnetic field vanishes (*B* = 0). In this case, the vector potential $\overrightarrow{A}=xB{\hat{e}}_{y}=0$ and the Hamiltonian for the quantum wire system in Eq. (1) can be written as

27 $$H=\frac{{p}_{x}^{2}+{p}_{y}^{2}}{2m}+\frac{1}{2}m{\omega }_{o}^{2}{x}^{2}+eFx+\frac{{\alpha }_{r}}{\hslash }[{\sigma }_{x}{p}_{y}-{\sigma }_{y}{p}_{x}]\mathrm{.}$$

The momentum component *p*_*y*_ can be replaced by *ℏk* because the Hamiltonian is translationally invariant along the y-direction. Eq. (27) with an external electric field *F* = 0 is similar to^34^

28 $$H=\frac{{p}_{x}^{2}}{2m}+\frac{1}{2}m{\omega }_{o}^{2}{x}^{2}+\frac{{\hslash }^{2}{k}^{2}}{2m}+\frac{{\alpha }_{r}}{\hslash }[\hslash k{\sigma }_{x}-{\sigma }_{y}{p}_{x}]\mathrm{.}$$

The Hamiltonian in Eq. (28) can be written using creation and annihilation operators of a shifted harmonic oscillator, $({a}^{\dagger })$ and (*a*), for a given *k* = 0 in the following dimensionless form

29 $$\frac{H}{\hslash {\omega }_{o}}=({a}^{\dagger }a+\frac{1}{2})+\frac{1}{2\sqrt{2}}\frac{{l}_{o}}{{l}_{so}}({a}^{\dagger }{\sigma }_{-}+a{\sigma }_{+}\mathrm{).}$$

**Figure 7:**
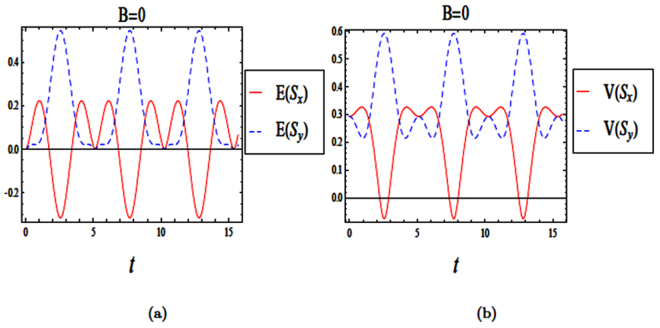
The time evolution of entropy and variance squeezing for a quantum wire with SOI in the absence of a magnetic field (*B* = 0) and *l*_*so*_ = *l*_*o*_. The initial states is an excited state, such that *θ* = 0 and the relative phase *ϕ* = 0. (**a**) Entropy squeezing factors *E*(*S*_*x*_) and *E*(*S*_*y*_); (**b**) Variance squeezing factors *V*(*S*_*x*_) and *V*(*S*_*y*_).

**Figure 8:**
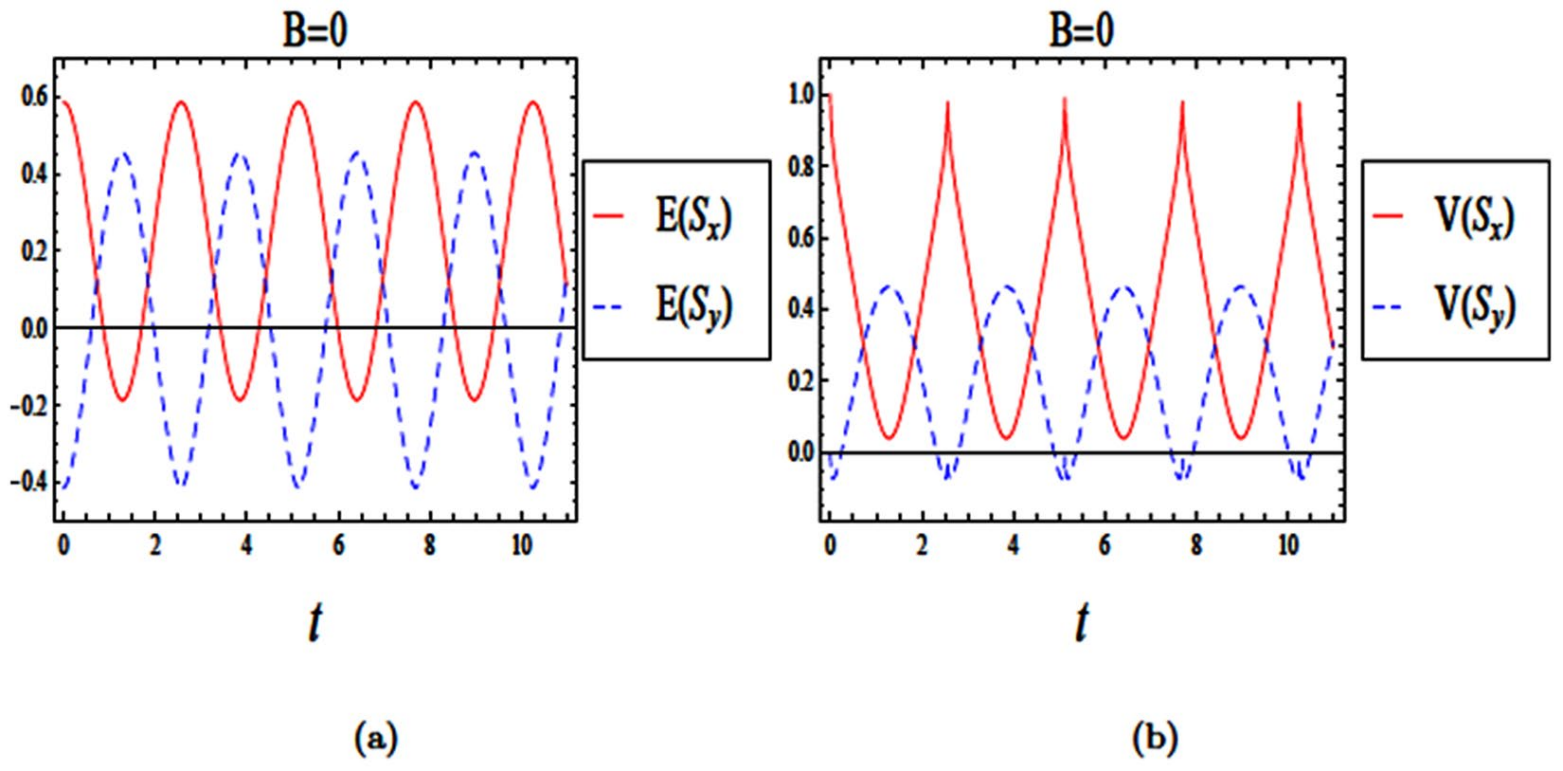
The time evolution of entropy and variance squeezing for a quantum wire with SOI in the absence of a magnetic field (*B* = 0) and *l*_*so*_ = *l*_*o*_ for a superposition state *θ* = *π*/2 and *ϕ* = *π*/2. (**a**) Entropy squeezing factors *E*(*S*_*x*_) and *E*(*S*_*y*_); (**b**) Variance squeezing factors *V*(*S*_*x*_) and *V*(*S*_*y*_).

**Figure 9:**
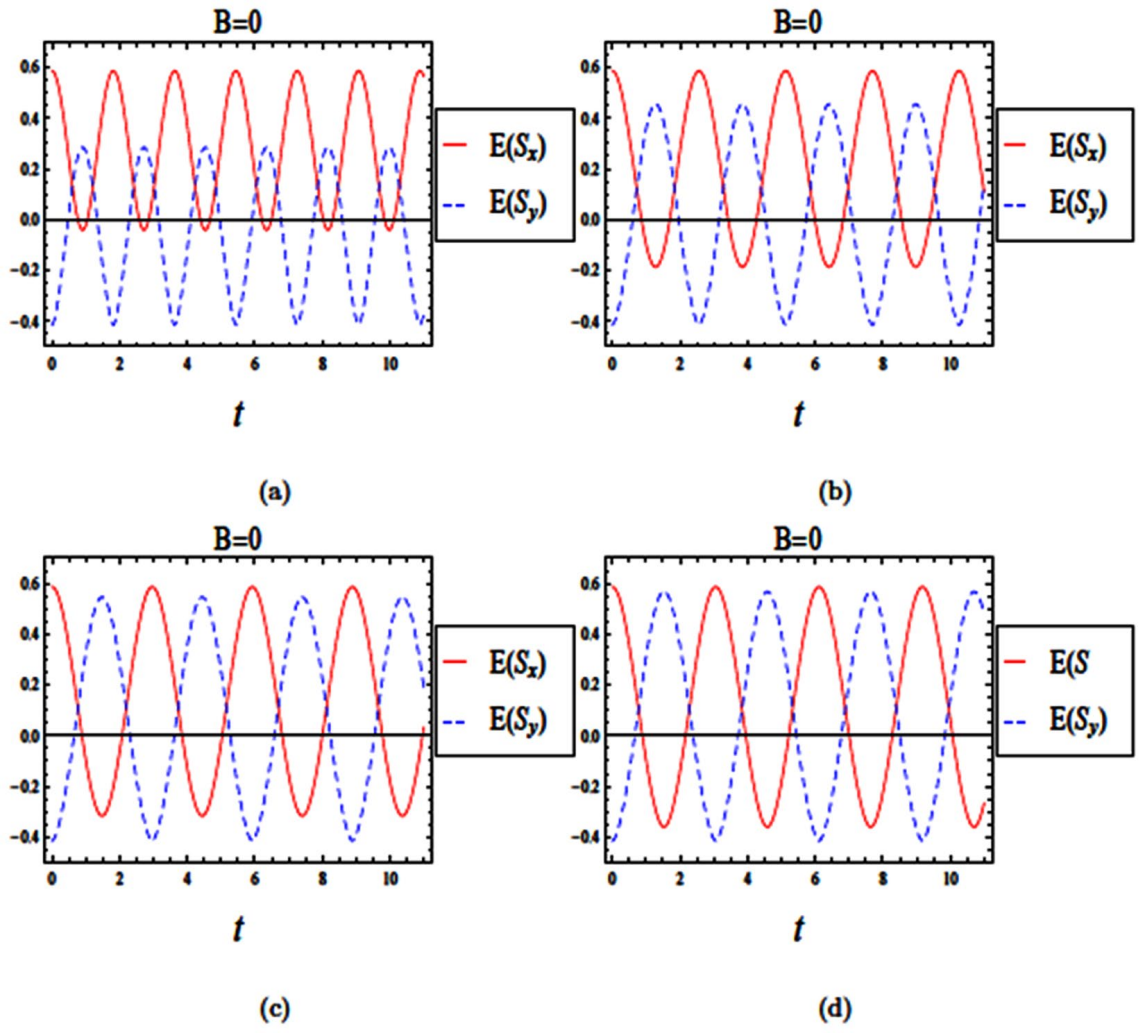
The time evolution of entropy squeezing *E*(*S*_*x*_) and *E*(*S*_*y*_) for a quantum wire with SOI in the absence of a magnetic field (*B* = 0) for a superposition state, *θ* = *π*/2, *ϕ* = *π*/2. (**a**) *l*_*so*_ = 0.5*l*_*o*_; (**b**) *l*_*so*_ = *l*_*o*_; (**c**) *l*_*so*_ = 2*l*_*o*_; (**d**) *l*_*so*_ = 3*l*_*o*_.

We have investigated the time evolutions of both entropy squeezing and variance squeezing in a quantum wire with SOI and with *B* = 0 and *l*_*so*_ = *l*_*o*_, when the initial states are either in an excited or a superposition state (see Figs (7) and (8)). Also, we plot the effect of spin-orbit interaction strength (*l*_*so*_) on entropy squeezing of a quantum wire without a magnetic field (*B* = 0)in Fig. (9). From Figs (7–9), we can conclude that the effect of spin-orbit interaction strength and the initial state of the system on the information entropy squeezing of a quantum wire without a magnetic field are similar to the results of a weak magnetic field in (4.2) and (4.3), as expected.

## Conclusion

In this paper, we have investigated the information entropy squeezing in a ballistic quantum wire with Rashba spin-orbit interaction in the presence of both strong and weak magnetic fields when the initial state is either in an excited or a superposition state. Our results show that the entropy squeezing is a more sensitive measure of information squeezing compared to variance squeezing. Furthermore, we have explored the effects of spin-orbit interaction strength and the initial state of the system on the information entropy squeezing for different strengths of the magnetic field. The results show that there is a strong relationship between the spin-orbit interaction and the strength of entropy squeezing. When the strength of the spin-orbit interaction is increased, the strength of the entropy squeezing is increased and vice versa. Additionally, there is a relationship between the initial state and the number of squeezed components. If the system starts in an excited state, the squeezing will only occur in one component or quadrature. On the other hand, when the system starts in a superposition state, squeezing can occur in both quadratures. Thus we have identified new ways to control the strength and the component of entropy squeezing in a nanowire system compared to previous work. This has potential applications in future quantum information technologies.
